# Anti-Plasmodial Polyvalent Interactions in *Artemisia annua* L. Aqueous Extract – Possible Synergistic and Resistance Mechanisms

**DOI:** 10.1371/journal.pone.0080790

**Published:** 2013-11-14

**Authors:** John O. Suberu, Alexander P. Gorka, Lauren Jacobs, Paul D. Roepe, Neil Sullivan, Guy C. Barker, Alexei A. Lapkin

**Affiliations:** 1 School of Life Sciences, University of Warwick, Coventry, United Kingdom; 2 Centre for Infectious Disease, Department of Chemistry, Georgetown University, Washington, District of Columbia, United States of America; 3 Sensapharm Ltd, Business and Innovation Centre, Sunderland, United Kingdom; 4 Department of Chemical Engineering and Biotechnology, University of Cambridge, Cambridge, United Kingdom; RWTH Aachen University, Germany

## Abstract

*Artemisia annua* hot water infusion (tea) has been used in *in vitro* experiments against *P. falciparum* malaria parasites to test potency relative to equivalent pure artemisinin. High performance liquid chromatography (HPLC) and mass spectrometric analyses were employed to determine the metabolite profile of tea including the concentrations of artemisinin (47.5±0.8 mg L^-1^), dihydroartemisinic acid (70.0±0.3 mg L^-1^), arteannuin B (1.3±0.0 mg L^-1^), isovitexin (105.0±7.2 mg L^-1^) and a range of polyphenolic acids. The tea extract, purified compounds from the extract, and the combination of artemisinin with the purified compounds were tested against chloroquine sensitive and chloroquine resistant strains of *P. falciparum* using the DNA-intercalative SYBR Green I assay. The results of these *in vitro* tests and of isobologram analyses of combination effects showed mild to strong antagonistic interactions between artemisinin and the compounds (9-epi-artemisinin and artemisitene) extracted from *A. annua* with significant (IC_50_ <1 μM) anti-plasmodial activities for the combination range evaluated. Mono-caffeoylquinic acids, tri-caffeoylquinic acid, artemisinic acid and arteannuin B showed additive interaction while rosmarinic acid showed synergistic interaction with artemisinin in the chloroquine sensitive strain at a combination ratio of 1:3 (artemisinin to purified compound). In the chloroquine resistant parasite, using the same ratio, these compounds strongly antagonised artemisinin anti-plasmodial activity with the exception of arteannuin B, which was synergistic. This result would suggest a mechanism targeting parasite resistance defenses for arteannuin B’s potentiation of artemisinin.

## Introduction

The use of *Artemisia annua* (*Qing Hao*) in traditional Chinese pharmacopeia includes the treatment of fevers and chills [1,2]. In the 1970s, the active principle in the extract was isolated and identified as artemisinin (1), a sesquiterpene lactone. The effectiveness of artemisinin is structurally due to the trioxane pharmacophore and the activation of the compound occurs via the cleavage of the endoperoxide bridge [3]. The mechanism for the activation of artemisinins and their interaction with the parasite are not fully understood. Different but not mutually exclusive mechanistic models have been proposed with evidence for and against each model [4]. A number of studies [5,6] have suggested that artemisinins act by heme dependent activation of the trioxane bridge in the parasites’ food vacuole to produce free radicals which then disrupt heme detoxification and therefore lead to parasite toxicity. This hypothesis and other alternative mechanisms for the mode of action of artemisinins have been studied and reviewed by several authors [3,4,7–13]. Artemisinin and its derivatives have now been established in various combination therapies (ACTs) as effective anti-malarial treatments against multidrug-resistant *P. falciparum* infection [14,15].

In some parts of Asia and Africa, a hot water infusion (tea) of the plant is used as a self-medication for malaria. The use of tea in this way has raised concern of the possible development of parasite resistance as a result of un-standardised use of artemisinin in these tea preparations [16]. Consequently, the World Heath Organisation (WHO) in a position statement has called for “extensive fundamental and clinical research” which demonstrates both efficacy and safety for the use of tea and other non-pharmaceutical forms of *A. annua* extract before recommendation for treating malaria [17]. 

The recipes in ancient Chinese texts for preparing *Qing Hao* extracts for the treatment of fevers include soaking, followed by wringing or pounding, followed by squeezing the fresh herb [1,2,18]. In their study, Rath et al. [19] found that adding boiling water to the leaves, stirring briefly and leaving covered for 10 minutes, then filtering and gently squeezing the leaves to release residual water gave the best extraction efficiency (86%) for artemisinin in the preparation, relative to the total amount of the compound in leaves. In the literature, a range of aqueous extraction efficiencies (25-90%) has been reported for artemisinin [19–21]. Due to the differences in the content of artemisinin in tea preparation, Van der Kooy and Verpoorte [21] quantified artemisinin in tea prepared by different methods. They observed that the extraction efficiency is temperature-sensitive and that efficiencies of above 90% are attainable.

**Figure 1:**
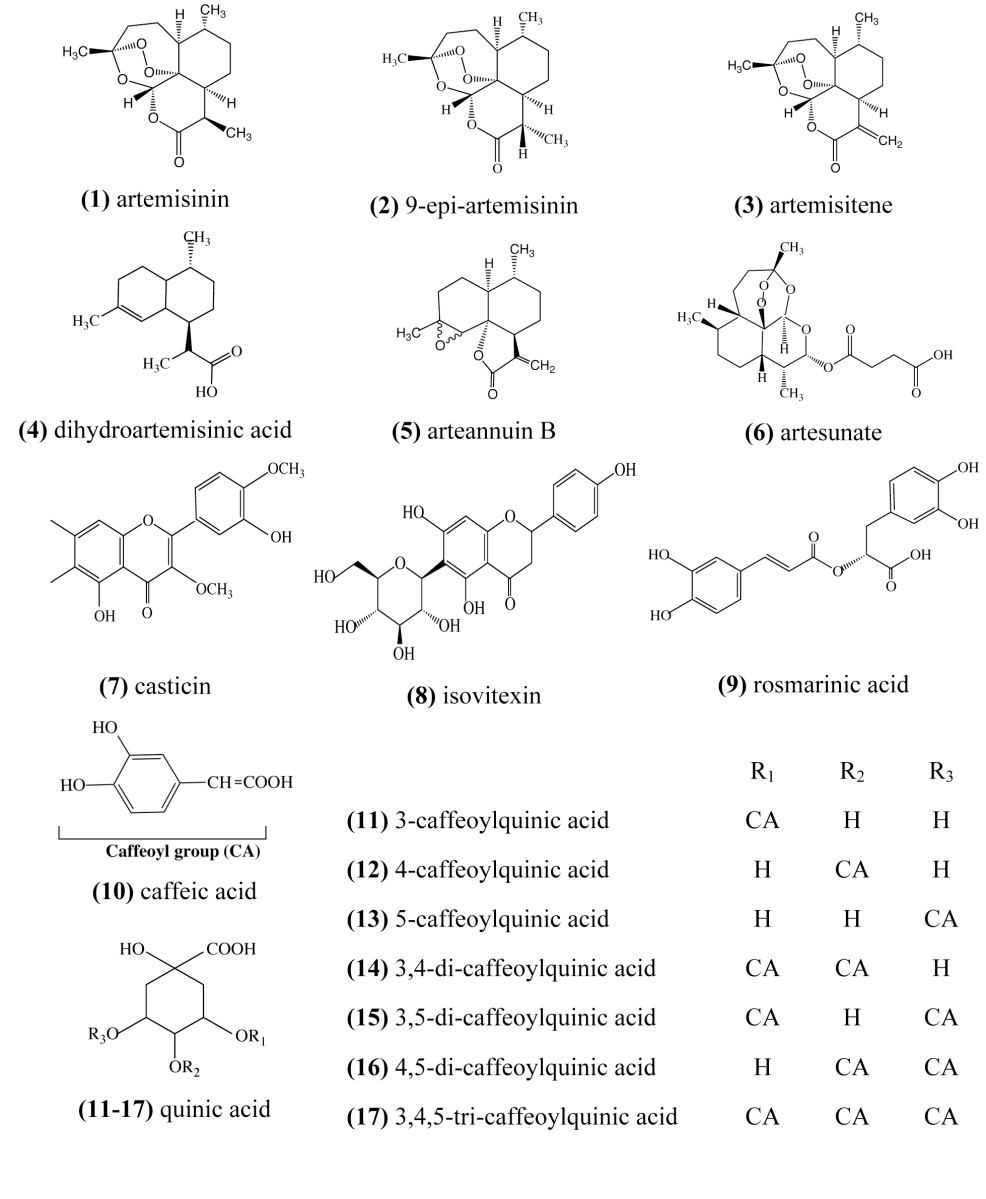
Structures of some artemisinin related compounds, flavonoids and acids identified in *A. annua* extract.

In some studies evaluating the activity of *A. annua* extracts, the amount of artemisinin in these extracts cannot fully account for its effectiveness against *Plasmodium* parasites *in vitro* and *in vivo* [16,19]. Mouton et al. however did not find any evidence of improved potency for their extracts relative to the artemisinin content [22]. Apart from artemisinin, there are around 30 other sesquiterpenes and over 36 ﬂavonoids identified in the plant (Figure 1), some of which have shown limited anti-plasmodial properties [23]. Five flavonoids, including casticin (7), have been shown to potentiate the activity of artemisinin [24,25]. Interestingly, the potentiating effect of these flavonoids was not observed with chloroquine (CQ). Billia et al. [26] observed that although these flavonoids have no effect on hemin (chloroferriprotoporphyrin IX) themselves, they do catalyse a reaction between artemisinin and hemin.

Weathers and Towler [27] have shown that poly-methoxylated flavonoids like casticin are poorly extracted and unstable in the aqueous tea infusion. This suggests that compounds other than this class of flavonoids are likely to be responsible for the reported improvement in the potency of artemisinin in tea infusion. A recent analysis by Cabonara et al. [28] of tea prepared from *A. annua* leaves by infusion in hot water for 1, 24 and 48 hours, identified a series of caffeoyl and feruloyl-quinic acids as main components of the infusion, together with some flavonoids. Chlorogenic or caffeoylquinic acids (CQAs) are esters of caffeic and quinic acids (Figure 1). They possess a broad spectrum of pharmacological properties, including antioxidant, hepato-protectant, antibacterial, anti-histaminic, chemo-preventive and other biological effects [29–32]. 

To our knowledge, only the interactions of artemisinin with the poorly extracted poly-methoxylated flavonoids found in *Artemisia* tea have been studied. This study therefore aims at understanding other possible interactions and mechanisms involved in artemisinin activity in the plant extract, and the effects of these interactions on parasite resistance to artemisinin.

## Materials and Methods

### 2.1: Chemicals

Reference standards of artemisinin (98%), rosmarinic acid, caffeic acid and casticin were obtained from Sigma-Aldrich (Dorset, UK). Dihydroartemisinic acid (> 96%) was purchased from Apin Chemicals (Oxfordshire, UK). 9-Epi-artemisinin (98%) was sourced from Sensapharm Ltd (Sunderland, UK). Artemisitene, artemisinic acid and arteannuin B were kindly provided by Walter Reed Army Institute of Research (Washington, DC, USA). The chlorogenic acids (>99%) and isovitexin (>99%) were obtained from Biopurify (China). LC-MS grade formic acid in water, acetonitrile and HPLC grade acetonitrile were obtained from Fisher Scientific (UK). Purified water (~18 MΩ cm^-1^) was dispensed from a Milli-Q system (Millipore, UK).

### 2.2: Plant materials

High yielding, dried *A. annua* biomass was obtained from BIONEXX Madagascar and stored under dark, cool conditions until use. 

### 2.3: Plant extracts

*A. annua* tea was prepared according to published methods with slight modification [1,33]. Briefly, 1 L of boiling water was added to 5 g of dried plant material, stirred and stored in the dark for 1 hour. The extract was filtered *in vacuo* and lyophilised after freezing to obtain the dried tea extract which was used in the *Plasmodium* assays and in metabolite profiling.

### 2.4: Sample preparation – solubility studies

The solubility of artemisinin, artemisitene and 9-epi-artemisinin in aqueous solvent at room temperature (22 °C) was determined by the method employed by Wang et al. [34], with modifications. A saturated solution was prepared by dissolving excess amount of the pure (> 99.0%) standard of each material in 1 mL de-ionised water (MS grade, Brucker, UK) and vortexed. This suspension was allowed to settle and the supernatant filtered through a 0.1 µm syringe filter (Fisher Scientific, UK). Appropriate volume of the filtrate was diluted with the mobile phase for mass spectrometry (MS/MS) analysis.

### 2.5: Mass spectrometry method for artemisinins

The method by Suberu et al. [35] was employed. Briefly, the MS/MS system was operated with an ESI interface in positive ionisation mode (ESI+). The cone and desolvation gas flow rates were set at 45 L h^-1^ and 800 L h^-1^, respectively. The MS parameters were automatically defined using Waters IntelliStart® software for the tuning and calibration of the tandem quardrupole analyser (TQD) and subsequently manually optimized for all analytes. Capillary voltage was set at 2.8 Kilovolts, collision voltage at 7 volts, source temperature was 150 °C and cone voltage was set at 24 volts. A multiple reaction-monitoring (MRM) transition of 283→219+229+247+265, 283→209+219+247+265, 281→217+227+245+263 for artemisinin, 9-epi-artemisinin and artemisitene was employed. Quantification was determined using MRM modes for the above transitions. The dwell time was automatically set at 0.161 seconds. Data were acquired by MassLynx v4.1 software and processed for quantification with QuanLynx v4.1 (Waters Corp., Milford, MA, USA). 

The high performance liquid chromatography (HPLC) system coupled to the mass spectrometer consisted of a binary pump, a cooling auto-sampler with an injection loop of 10 µL set at 10 °C. The column heater was set at 30 °C and a Genesis® Lightn C18 column (100 × 2.1 mm; 4 μm) (Grace, IL, USA) protected by an Acquity-LC column in-line filter unit (0.2µm in-line frit) was used for separation of metabolites. The mobile phase consisted of A: 0.1% formic acid in water and B: 0.1% formic acid in acetonitrile used in the following gradient: 0–7.00 min, 25-98% B; 7-9.5 min, 98% B; 9.5-10 min, 98-25% B; 10-15 min, 25% B; at a flow rate of 0.4 mL min^-1^. Weak wash solvent was 10% acetonitrile, strong and needle wash solvent was a mixture of acetonitrile, propan-2-ol, methanol and water (30:30:30:10 v/v/v/v).

### 2.6: HPLC method for acids and flavonoid

Analysis of acids and flavonoid was performed on an Agilent 1100 series HPLC equipped with a quaternary pump, auto-sampler, photodiode array (PDA) and a degasser. The chromatographic method by Carbonara et al. [28] was used in the analysis with slight modifications. Briefly, the solvent system consisted of A (0.1% acetic acid, brought to pH 4 with NaOH) and B (0.1% acetic acid in acetonitrile) using a gradient elusion of 0-60 min: 12-25% B, 60-80 min: 25-60% B, 80-85 min: 60-100% B. The system was equilibrated back to 12% B for 5 minutes before the next run. Analytes were separated and resolved at a flow rate of 1 mL min^-1^ on a Phenomenex Luna C18 column (250 mm x 4.60 mm, 5 µm particle size) attached to a C18 guard column. Detection and quantification was at 310 nm for caffeic acid, chlorogenic acids and isovitexin. Rosmarinic acid was analysed at 330 nm wavelength. 

### 2.7: *Plasmodium* assay

Determination of 50% growth inhibitory concentration (IC_50_) values of extracts, compounds and combinations against CQ-sensitive (CQS; HB3) and CQ-resistant (CQR; Dd2) strains of *P. falciparum* was performed at Georgetown University, Washington, DC, USA, using a previously reported protocol [36] with minor modifications. Typically, test samples were dissolved in DMSO to give a stock solution, followed by serial dilution using complete media (RPMI 1640 supplemented with 10% (v/v) type-O^+^ human serum, 25 mM HEPES (pH 7.4), 23 mM NaHCO_3_, 11 mM glucose, 0.75 mM hypoxanthine and 20 µg/L gentamicin) to generate working stocks. 100 μL of these stock solutions were transferred into pre-warmed (37 °C) 96-well plates. 100 μL of asynchronous parasite culture at 2% parasitemia, 4% hematocrit was transferred into each drug (*A. annua* plant extract) pre-loaded well, for a final 1% parasitemia, 2% hematocrit. The final concentration of DMSO was 2.5%. Plates were transferred to a gassed (90% N_2_, 5% O_2_, 5% CO_2_) airtight chamber and incubated at 37 °C for 72 hours. Following this incubation, 50 μL of 10X SYBR Green I dye (diluted with complete media from a 10000X concentrate in DMSO) was added to each well and plates incubated for an additional 1 hour at 37 °C to allow DNA intercalation. Fluorescence was measured at 530 nm (490 excitation) on a Spectra GeminiEM plate reader (Molecular Devices, USA). IC_50_ values were obtained from sigmoidal curves fit of parasite growth vs. drug concentration using SigmaPlot 10.0, and are the average of three replicates. CQ was included as a positive control in the assay.

### 2.8: Combination analysis

Interactions between compounds were evaluated by isobologram analysis [37,38]. Briefly, a master stock solution is prepared for each compound such that its concentration following four or five twofold dilutions approximates the IC_50_. These stock solutions were mixed at ratios of 0:4, 1:3, 1:1, 3:1 and 4:0 (v/v) to give working combination stocks. Subsequently, the combination stocks were twofold serially diluted to generate a full dose concentration range for each v/v mixture, which were then analysed under standard growth inhibitory assay conditions (see above) to provide dose response curves and an IC_50_, for each component of each v/v mixture.

### 2.9: Data analysis for in vitro combination studies

IC_50_ values for each compound alone and in the combination were used to calculate FICs (fractional inhibition concentrations) as described elsewhere [39,40]. The FICs were summated to obtain the fractional inhibition concentration index (FIC_index_) for the combination as in the equation below:

$$FIC_{index}=FIC_{A}+FIC_{B}$$

where:

$$FIC_{A}=\frac{IC_{50}\;of\;Drug\;A\;in\;Combination}{IC_{50}\;of\;Drug\;A\;Alone}$$

$$FIC_{B}=\frac{IC_{50}\;of\;Drug\;B\;in\;Combination}{IC_{50}\;of\;Drug\;B\;Alone}$$

The following categorisation was used to determine the type of interactions between compounds evaluated: synergy (FIC_index_ <0.9), additivity (0.9<FIC_index_<1.5) and antagonism (FIC_index_ >1.5) [39,40].

## Result and Discussion

### 3.1: Composition of *A. annua* tea

Table 1 shows the metabolites in the aqueous extract analysed by both MS/MS and HPLC methods and their quantities in milligrams per litre of extract. The compounds analysed were based on the in extenso analysis by Carbonara et al. [28], who showed them to be among the major metabolites (quantitatively) in *A. annua* tea infusions. Some of these metabolites (like 3-caffeoylquinic acid) also have important dietary profiles [41,42]. In addition, artemisinin-related compounds, which we have previously detected in such extracts, were also analysed. The level of artemisinin reported [2,19,21,28,43] for tea extract is varied and the values obtained in this study (47.5 mg L^-1^) are within the reported range. These could be due to variation in biomass and the tea preparation method that was employed, but might also be due to differences in the biomass-to-solvent ratio used. Carbonara et al. [28] used a solvent to biomass ratio of 26:1 (v/w), while this study, as well as others [19,21], employed the therapeutically recommended ratio (200:1, v/w or 5 g L^-1^) [44].

**Table 1:** Metabolites in the aqueous *A. annua* extract analysed by both MS/MS and HPLC methods quantified as milligrams per litre of tea.

**Compound**	Amount (mg L^-1^ of tea)*
Artemisinin	47.5±0.8
Arteannuin B	1.3±0.0
Dihydroartemisinic acid	70.0±0.3
Caffeic acid	0.8±0.00
3,5-Di-caffeoylquinic acid	57.0±1.7
3-Caffeoylquinic acid	72.0±1.6
4-Caffeoylquinic acid	20.4±1.6
4,5-Di-caffeoylquinic acid	31.6±4.0
5-Caffeoylquinic acid	9.0±0.7
Isovitexin	105.0±7.2
Rosmarinic acid	1.1±0.0

* Values are an average of triplicate determinations with ± S.E.M.

Dihydroartemisinic acid (**4**) (70 mg L^-1^) and arteannuin B (**5**) (1.3 mg L^-1^) are the only biosynthetic precursors of artemisinin detected in the tea extract using our method [35]. Therefore artemisinin is the only compound among the metabolites we analysed in the tea with significant (IC_50_ <1 μM) anti-plasmodial activity (Table 2).

**Table 2:** IC_50_ of extracts and components of *A. annua* in CQ-sensitive (HB3) and resistant (Dd2) strains.

	**IC_50_ (nM)^a^**	
Compound/extracts	HB3 strain	Dd2 strain
Chloroquine (CQ)	21.8 ± 2.4	202.9 ± 10.7
Artemisinin	22.6 ± 0.7	21.2 ± 2.3
Artesunate	8.8 ± 0.3	5.6 ± 0.6
Artemisitene	88.4 ± 9.9	74.1 ± 7.8
9-epi-artemisinin	59.2 ± 1.7	62.2 ± 1.0
Artemisia aqueous extract (Tea)^b^	7.6 ± 3.4	2.9 ± 0.4
	**IC_50_ (µM)^a^**	
Artemisinic acid	77.8 ± 1.5	61.6 ± 7.5
Arteannuin B	3.2 ± 0.1	4.8 ± 0.4
Dihydroartemisinic acid	21.1 ± 0.7	17.7 ± 4.2
Caffeic acid	60.4 ± 4.3	47.5 ± 8.8
3-Caffeoylquinic acid	69.4 ± 6.4	61.4 ± 4.3
4-Caffeoylquinic acid	61.4 ± 4.3	53.6 ± 5.0
5-Caffeoylquinic acid	84.8 ± 6.4	85.3 ± 4.2
3,4-Caffeoylquinic acid	36.2 ± 1.0	49.0 ± 6.8
4,5-Caffeoylquinic acid	29.3 ± 2.4	43.2 ± 4.2
3,4,5-Caffeoylquinic acid	181.4 ± 2.1	88.2 ± 6.2
Rosmarinic acid	65.1 ± 5.0	65.0 ± 7.0
Isovitexin	72.5 ± 6.8	48.1 ± 4.5
Casticin	17.9 ± 4.7	12.2 ± 1.8

^a^ IC_50_ values are an average of at least three independent measurements each performed in triplicate, and are shown ± S.E.M of the three independent experiments. ^b^ IC_50_ of extract determined based on the artemisinin content (i.e. ART IC_50_ of extract) see Table 2.

3-Caffeoylquinic acid (**11**) was found to be the most abundant (72 mg L^-1^) of the caffeic derivatives (**11-17**) in the analysed extract, followed by 3,5-di-caffeoylquinic acid (**15**) (57 mg L^-1^). Caffeic acid (**10**) was the least abundant (0.8 mg L^-1^) of the evaluated acids. Isovitexin (**8**) was the only flavonoid analysed (105 mg L^-1^), being relatively abundant in the extract. Some classes of flavonoids have poor aqueous solubility and limited profiles of these compounds in aqueous extract have been reported [27,28]. Lower level of rosmarinic acid (**9**) (1.1 mg L^-1^) was detected in our samples, compared to the levels found by De Magalhaes et al [43]. However, widely different concentrations of the acid were reported in the cultivars and samples they analysed. The acid was not detected in the analysis by Carbonara et al [28]. Van der Kooy and Verpoorte [21] have also shown that the method employed in preparing the hot water infusion does affect the amount of artemisinin and therefore other co-metabolites extracted. These differences in profiles and concentration levels of metabolites seem to suggest that composition of prepared tea infusions differ and is significantly influenced by method of preparation and the *Artemisia* cultivar used.

### 3.2: Anti-*plasmodium extracts* and bioactive compounds in *A. annua*

Table 2 shows IC_50_ anti-plasmodial values for pure compounds and extracts of *A. annua* plant. Between three- and seven-fold potentiation of artemisinin activity was observed for *A .annua* aqueous (tea) extract in CQ-sensitive (HB3) and CQ-resistant (Dd2) strains respectively. Only artemisitene (3) (IC_50_, 88.4±9.9/74.1±7.8 nM, HB3/Dd2) and 9-epi-artemisinin (2) (IC_50_, 59.2±1.7/62.2±1.0 nM, HB3/Dd2) showed significant anti-plasmodial activities (IC_50_ <1 µM) among the artemisinin biosynthetic precursors evaluated. 9-Epi-artemisinin and artemisitene respectively showed about one third and one fourth of the activity of artemisinin. Acton et al. [45] observed a similarly reduced activity for 9-epi-artemisinin and artemisitene, compared to artemisinin in D6 and W2 strains of *P. falciparum*. Artemisinin has a chiral molecular structure and the bioactivity of the molecule is influenced by its absolute configuration.

To investigate if solubility of these artemisinin analogues could be partially responsible for the reduced activity, we determined the aqueous solubilities of artemisinin, artemisitene and 9-epi-artemisnin. Table 3 shows the solubility of these compounds at experimental conditions.

**Table 3:** Solubility of artemisinin, artemisitene and 9-epi-artemisinin in water at 22 °C and atmospheric pressure.

Compound	Solubility [mg L^-1^]* at 22 °C
Artemisinin	74.27±2.10
Artemisitene	74.21±2.99
9-Epi-artemisinin	133.08±5.44

* Values are an average of triplicate determinations with ± S.E.M.

Under these conditions, 9-epi-artemisinin has a higher solubility, about twice that of artemisinin or artemisitene. The lower bioactivity could not be explained based on the solubility data alone, although the experimental data was obtained at 22 °C (Table 3). We do not expect the pattern observed to change significantly at physiological conditions.

Woerdenbag et al. [46] observed that the anti-cancer activity of 11-hydroxy-11-epi-artemisinin (C11 in older and C9 in newer references for the structure) was about threefold less than the conformer, which is the same threefold difference we observed in the anti-plasmodial activity for epimerisation at C9 (Table 2). If the threefold activity difference is consistent regardless of the differences in molecular targets and effect, this may suggest a common upstream differentiation point of molecule activation. The lower activity of 9-epi-artemisinin may therefore be due in part to a structural conformation that is relatively more difficult to activate compared to artemisinin. 

### 3.3: Antagonism of artemisinin with biosynthetic precursors

**Figure 2:**
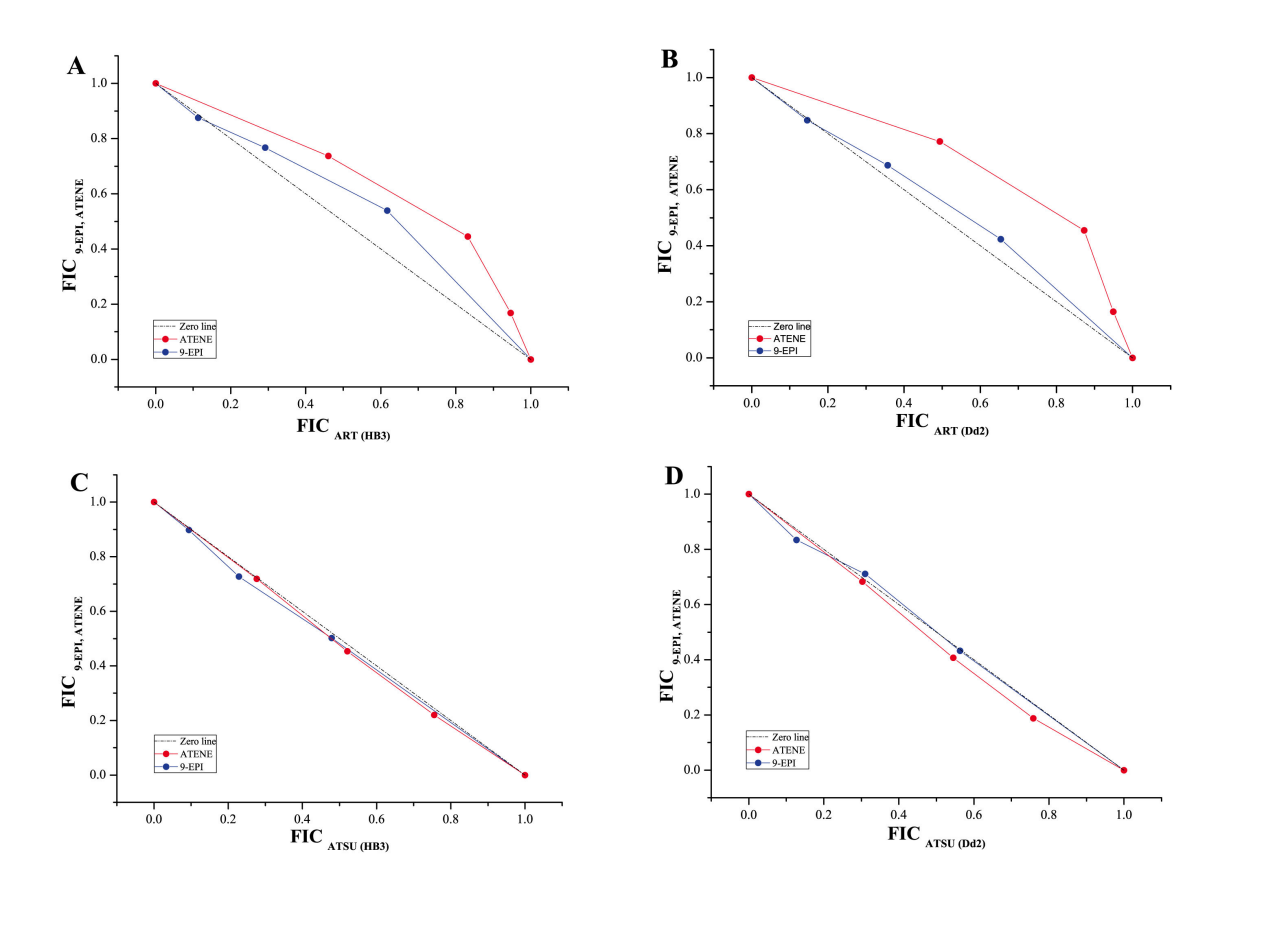
Isobologram showing the plot of fractional inhibitory concentration (FIC) of 9-epi-artemisinin (EPI) and artemisitene (ATENE) against FIC of artemisinin (ART) and artesunate (ATSU). Panel A - interaction of EPI and ATENE with ART in chloroquine-sensitive (CQS) HB3 strain. Panel B – same as in A but in CQ-resistant (CQR) Dd2 strain. Panel C - interaction of EPI and ATENE with ATSU in HB3. Panel D – same as C but in Dd2 parasite.

Figure 2 shows the interaction of artemisitene and 9-epi-artemisinin with artemisinin and artesunate (**6**). These biosynthetic precursors of artemisinin have significant (IC_50_ <1 µM) anti-plasmodial activities (Table 2). The interaction of artemisinin with 9-epi-artemisinin and artemisitene was antagonistic, but the interaction of these compounds with artesunate was additive in both chloroquine sensitive (HB3) and resistant (Dd2) strains.

The reason for the observed antagonistic interaction with artemisinin at the combinations investigated is unclear. Structurally, artemisinin, 9-epi-artemisinin and artemisitene are differentiated at C9. The difference from artemisinin is epimerisation of the methyl group for 9-epi-artemisinin and a methylene group attached instead for artemisitene (Figure 1). Given the minor structural differences, it is likely that these compounds have identical molecular targets and therefore possibly compete for these when combined. Conversely, due to the relatively large difference in structure and mass of artesunate and 9-epi-artemisinin or artemisitene, these compounds, when combined, may act on the same targets as well as on different molecular targets with the possibility of positive polyvalent interaction. Similarly, Wagner [47–49] has reported an *in vitro* synergistic inhibitory effect upon combining ginkgolides A and B from *Ginkgo biloba* extract for PAF-induced thrombocyte-aggregation. The difference between ginkgolide A and B is an oxygen atom (16 Da).

### 3.4: Analysis of other combinations

Table 4 shows the interaction of co-metabolites in *A. annua* extracts with artemisinin. In the CQ-sensitive (HB3) strain, 3-caffeoylquinic acid (3CA) showed additive interaction at 1:3 (v/v), which became synergistic at higher ratio of the acid to artemisinin (1:10, 1:100 v/v). For casticin, the interaction at 1:3 (artemisinin to casticin, v/v) is antagonistic. Synergistic interaction is however reported [24,25] for combination ratios at the range of 1:10-1000 (artemisinin to casticin, v/v). 

**Table 4:** Anti-plasmodial interactions of co-metabolites with artemisinin in CQ-sensitive (HB3) and CQ-resistant (Dd2) strains.

	HB3		Dd2	
Combination	FIC_index_	Interaction	FIC_index_	Interaction
1:3 ART:CA	1.570	Antagonistic	4.046	Antagonistic
1:3 ART:3CA	1.172	Additive	2.088	Antagonistic
1:10 ART:3CA	0.685	Synergistic	1.087	Additive
1:100 ART:3CA	0.781	Synergistic	1.177	Additive
1:3 ART:4CA	1.088	Additive	4.266	Antagonistic
1:3 ART:5CA	0.928	Additive	2.460	Antagonistic
1:3 ART:34CA	2.253	Antagonistic	4.862	Antagonistic
1:3 ART:35CA	2.312	Antagonistic	4.749	Antagonistic
1:3 ART:45CA	2.315	Antagonistic	4.844	Antagonistic
1:3 ART:TCA	1.220	Additive	3.041	Antagonistic
1:3 ART:ISO	1.534	Antagonistic	4.829	Antagonistic
1:3 ART:CAS	1.921	Antagonistic	3.034	Antagonistic
1:3 ART:ATCID	1.467	Additive	4.152	Antagonistic
1:3 ART:ARTB	1.250	Additive	0.342	Synergistic
1:3 ART:RA	0.890	Synergistic	4.952	Antagonistic
1:3 ART:DHAA	1.801	Antagonistic	2.861	Antagonistic
1:3 ART:ATENE	3.480	Antagonistic	7.002	Antagonistic
ART	1	-	1	-

Art = artemisinin, CA = caffeic acid, 3CA = 3-caffeoylquinic acid, 4CA = 4-caffeoylquinic acid, 5CA = 5-caffeoylquinic acid, 3,4 CA = 3,4-di-caffeoylquinic acid, 3,5CA = 3,5-di-caffeoylquinic acid, 4,5CA = 4,5-di-caffeoylquinic acid, TCA = 3,4,5-tri-caffeoylquinic acid, ISO = siovitexin, CAS = casticin, ATCID = artemisinic acid, ARTB = arteannuin B, RA = rosmarinic acid, DHAA = dihydroartemisinic acid, ATENE = artemisitene.

Therefore, using the FIC index of casticin (1.9) as a benchmark for potential positive interactions, compounds like isovitexin, caffeic acid and dihydroartemisinic acid that show antagonistic interactions at 1:3 may also, like casticin, interact synergistically at a higher ratio. Rosmarinic acid was synergistic at a 1:3 combination with artemisinin (v/v) and some chlorogenic acids were additive at this combination also. These compounds showing positive interactions with artemisinin may collectively be responsible for the potentiation of artemisinin in the tea extract. However, arteannuin B and artemisinic acid are poorly extracted in the aqueous extract.

**Figure 3:**
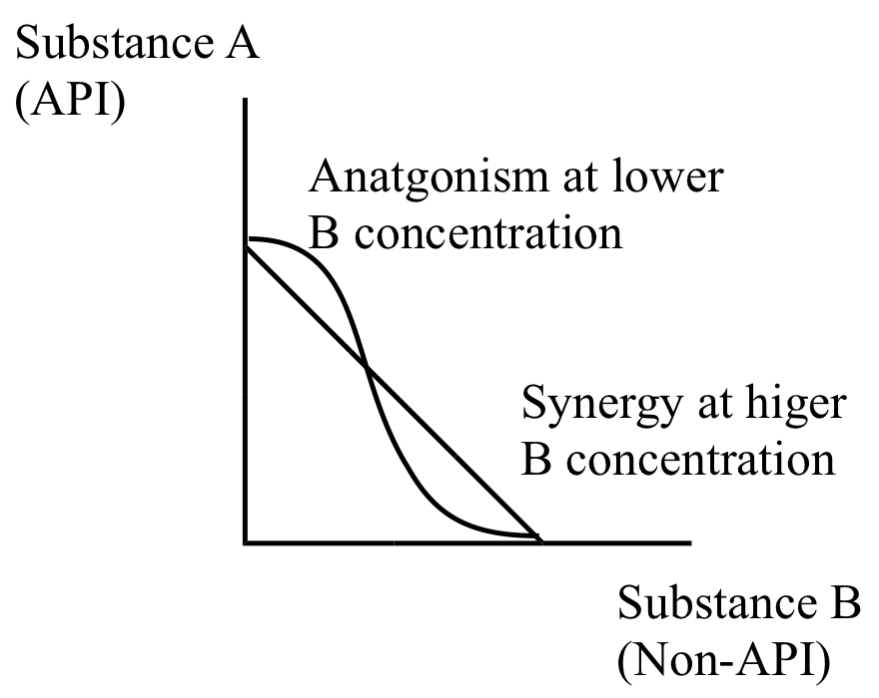
A schematic isobologram of the interaction of artemisinin (API) with anti-oxidant synergist (Non-API).

Casticin and 3-caffeoylquinic acid (3CA) are polyphenolic compounds that are natural anti-oxidants. Anti-oxidants at cellular redox sites are considered a “double edged sword” able to act either as anti-oxidant or pro-oxidant depending on conditions, such as dosage levels and presence of metal ions [50,51]. This “double edged sword” characteristic of anti-oxidant polyphenols could help explain our observation. At a lower combination with artemisinin, casticin and 3CA were anti-oxidative towards the ROS and carbon-centred radicals formed from artemisinin activation and, as a result, countered artemisinin activity *in vitro*. Conversely, at a higher concentration ratio to artemisinin, casticin and 3CA were pro-oxidative, enhancing the oxidative stress resulting from artemisinin’s activation, leading to improvement in artemisinin’s potency. A schematic isobologram to describe the interaction between an active pharmaceutical ingredient (API) like artemisinin (A) and synergists like casticin and 3CA (B, non-API) is shown in Figure 3.

### 3.5: Possible role of anti-oxidant defence network in resistance

Rosmarinic acid at the combination ratio evaluated had a potentiating effect (FIC_index_ 0.89) on artemisinin in the CQ-sensitive (HB3) strain (Table 4) but this effect was not reproduced in the resistant (Dd2) strain; rather a strong antagonistic effect (FIC_index_ 4.95) was observed. The effect of rosmarinic acid on artemisinin’s ability to mitigate the resistance mechanism of the parasite could be partly explained by the finding of Cul et al. [52] and others [53] who observed that *in vitro* resistance in *P. falciparum* is associated with increased *pfmdr-1* copy number and anti-oxidant activity. Some experiments with rosmarinic acid have reported strong anti-oxidant activity for the compound that is over three times that of trolox [54–56]. In the presence of rosmarinic acid, anti-oxidant activity may further be elevated thereby promoting increased resistance. A similar trend of activity in sensitive and resistant parasite strains in combination with artemisinin was observed for caffeic acid, 4-caffeoyl-quinic acid (12) and isovitexin with reported anti-oxidant properties [57–59]. This supports the possible role of the anti-oxidant defence network in parasite resistance to artemisinin [60]

### 3.6: Arteannuin B selectively potentiates the activity of artemisinin against parasite defence system

Arteannuin B at 3:1 (v/v) combination with artemisinin showed additive or no interaction (FIC_index_ 1.25) in the CQ-sensitive strain and a synergistic interaction (FIC_index_ 0.34) in the resistant parasite strain (Table 4). This is about a three-fold improvement in artemisinin’s potency against CQ-resistant *P. falciparum*. This is not reproduced in the CQ-sensitive strain. The potentiation of artemisinin by arteannuin B seems to be selectively directed at the parasites’ chloroquine resistance mechanism. This combination could therefore help to better understand the mechanism(s) involved in parasite defence network. Reproducing this three-fold improvement in potency with other artemisinin analogues could also help in the development of therapeutics effective against emerging drug-resistant strains. 

Arteannuin B is an unusual α-methylene-γ-lactone, transfused via a tertiary hydroxyl group [61]. This structure could account for its easy fragmentation/ionisation observed in mass spectrometry and reported facile rearrangement in acidic conditions [35,62]. 

## 4: Conclusions

In this study we examine interactions between artemisinin and co-metabolites found in *A. annua* plant extracts for chloroquine sensitive (CQS; HB3) and resistant (CQR; Dd2) *P. falciparum* malarial parasites. The aqueous extract (tea) showed about three to seven-fold potentiation in the parasite strains. When pure compounds were combined, 9-epi-artemisinin and artemisitene interacted antagonistically with artemisinin at the combinations evaluated. 9-epi-artemisinin and artemisitene were the only artemisinin-related metabolites with significant anti-plasmodial activity (IC_50_ <1 μM) among those evaluated. In CQS parasites, caffeic acids and their chlorogenic acid derivatives showed additive interactions with artemisinin at the combination ratio evaluated. 3-Caffeoylquinic acid’s interaction with artemisinin turned synergistic with the increased ratio of the former in the combination. Rosmarinic acid showed synergistic interaction with artemisinin in the drug sensitive strain but the interaction with artemisinin in the drug resistant strain was strongly antagonistic at the same level of combination. This antagonistic interaction in CQR parasites was also observed for caffeic acid and some of its derivatives known to have anti-oxidant properties. The observation supports literature evidence [52,53] for a potential role of anti-oxidants in parasite drug resistance. Therefore the effect of dietary anti-oxidants on artemisinin combination therapies used in the management of drug resistant *P. falciparium* malaria may need to be further investigated. 

Arteannuin B was found to selectively potentiate the activity of artemisinin in Dd2 parasites, suggesting some interaction with the CQR mechanism, since the potentiation of artemisinin by arteannuin B was not reproduced in CQS parasites. As a result of this specificity, arteannuin B could potentially be used as a probe to better understand parasite drug resistance mechanisms and the combination might prove useful for treating CQR strains of malaria. 
