# Correction: Anti-Plasmodial Polyvalent Interactions in *Artemisia annua* L. Aqueous Extract – Possible Synergistic and Resistance Mechanisms

**DOI:** 10.1371/annotation/57ae25b0-d2c8-444b-ab62-f047c5f3e01e

**Published:** 2013-11-25

**Authors:** John O. Suberu, Alexander P. Gorka, Lauren Jacobs, Paul D. Roepe, Neil Sullivan, Guy C. Barker, Alexei A. Lapkin

This article was republished on November 25, 2013 because of missing figures. Please download this article again to view the figures. 

